# Development of the clinical trial site performance metrics instrument: A study protocol

**DOI:** 10.1016/j.mex.2025.103165

**Published:** 2025-01-08

**Authors:** Mattia Bozzetti, Rosario Caruso, Silvia Soncini, Monica Guberti

**Affiliations:** a Department of Biomedicine and Prevention, University of Rome, Tor Vergata, Italy; b Health Professions Research and Development Unit, IRCCS Policlinico San Donato, San Donato Milanese, Italy; c Department of Biomedical Sciences for Health, University of Milan, Milan, Italy; d Azienda USL-IRCCS di Reggio Emilia, EBP & Research Unit of Health Profession, Reggio Emilia, Italy; e Nursing and Allied Health Professions, IRCCS Istituto Ortopedico Rizzoli, Bologna, Italy

**Keywords:** Clinical trials, Risk based monitoring, Central monitoring, Site performance, Performance metrics, Trial management, Development and Validation of a Clinical Trial Site Performance Measure (CT-SPM)

## Abstract

Clinical trials (CTs) are essential for medical advancements, yet their increasing complexity and cost demand improved efficiency in trial management. One major challenge in multicenter studies is the inconsistency in evaluating site performance. This study aims to develop and validate a Clinical Trial Site Performance Measure (CT-SPM) to assess “good performance” across trials. The tool will be tested and refined through psychometric analysis, resulting in both a comprehensive scale and a short form for universal application. The study is conducted in three phases: Phase 1 involves metric selection through expert consensus; Phase 2 focuses on psychometric testing to evaluate the reliability and validity of the instrument; and Phase 3 defines a cut-off for “good performance” using statistical models. This protocol aims to standardize site performance evaluation, potentially reducing research costs and enhancing trial quality.

• The study develops and validates a Clinical Trial Site Performance Measure (CT-SPM) using expert consensus and psychometric testing.
• A comprehensive and short-form tool will be created to evaluate site performance in multicenter clinical trials.
• A cut-off for “good performance” will be established using statistical models, facilitating consistent and efficient site evaluations.

## Graphical abstract

**Figure:**
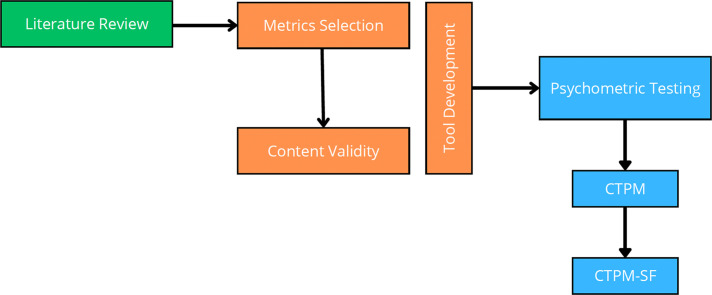

Specifications table

**Table:** 

Subject area:	Medicine and Dentistry
More specific subject area:	Clinical Trials and Research Performance Evaluation
Name of your protocol:	Development and Validation of a Clinical Trial Site Performance Measure (CT-SPM)
Reagents/tools:	Not applicable.
Experimental design:	This study will develop a Clinical Trial Site Performance Measure (CT-SPM) through three phases: 1) expert consensus and literature review to select key performance metrics; 2) psychometric testing (exploratory and confirmatory factor analysis) to validate the tool; 3) establishment of a performance cut-off using statistical modeling (ROC analysis).
Trial registration:	Not applicable.
Ethics:	This study involves the evaluation of clinical trial site performance metrics and does not involve direct research with human subjects or animals. Therefore, no informed consent or ethical approval specific to human or animal subjects is required.
Value of the Protocol:	• Provides a standardized tool (CT-SPM) to assess clinical trial site performance, helping to improve trial efficiency and data quality. • The tool offers both a comprehensive and short form, making it versatile for diverse clinical trial settings. • Establishes a clear cut-off for “good performance,” aiding in the early detection of site deviations from protocol, reducing trial delays, and costs.

## Background

Clinical trials (CTs) are intricate and resource-intensive projects that are critical to advancing medical knowledge and patient care. [1]. Clinical trials provide advantages to both society and people by generating knowledge and enhancing care, with some regarding participation in a clinical trial as the optimal management for cancer patients [2]. In recent years, CTs have exhibited a tendency towards increased complexity and expenses. This is mainly propelled by a growing consortia of stakeholders requiring additional outcomes, a wider range of patient populations, and adherence to rigorous regulatory standards [3,1]. Enhancing the efficiency and quality of trial execution is crucial for patients, sponsors, researchers, health professionals, and policymakers [4,5]. One of the biggest threats to their success is how well trial sites perform in terms of enlisting and keeping participants, as well as timely, thorough data collection. It is possible to increase the effectiveness and efficiency of the oversight of research conduct by standardizing the collecting, reporting, and monitoring of site performance data [[6], [7], [8]].

Despite regulatory encouragement, such as the Harmonisation of Technical Requirements for Pharmaceuticals for Human Use (ICH) GCP E6 (R2) guidance [9], practical implementation of RBM remains limited due to a lack of standardized metrics and empirical evidence supporting their effectiveness [8,10]. On-site monitoring is a crucial component of CT monitoring, involving the selection of specific criteria to evaluate the integrity of trial data and the safety of participants [10]. Metrics are numeric measurements used to evaluate a site's risk or performance [8]. Performance metrics have the potential ability to lower the financial cost of running a multicenter study as well as the research waste and delays in scientific advancement that take place when studies do not recruit enough participants, are poorly run, or have insufficient data collected [8]. While monitoring performances has been defined and is being encouraged by the regulators [9], little detail regarding the practical implementation of risk-based monitoring (RBM) exists [10] due to a scarcity of research that apply these measures and report on their impacts, as well as how locations conduct informal assessments [8]. Consequently, researchers may concentrate on excessive or uninformative indications. This study aims to develop and test a proximity index of “good performance” in research through the creation of two distinct tools. First, a comprehensive scale will be developed to include all relevant performance metrics, allowing each research coordinator to select and measure those metrics that are applicable and detectable within their specific site. This site-level customization ensures that the chosen metrics reflect the unique operational capabilities, patient populations, and resource availability of each location. By empowering research coordinators to tailor the metrics to their specific contexts, the approach promotes more accurate data collection and meaningful insights. Additionally, this methodology enhances site engagement and accountability, fostering a sense of ownership and commitment to the successful implementation of performance measurement initiatives. To complement this, a short form will be created to identify only the essential and universally applicable metrics across all CTs. This short form will serve to establish a cut-off point that dichotomously determines whether a study has substantially adhered to the protocol or experienced significant deviations.

## Description of protocol

This study will be conducted in three phases: the first phase focuses on tool design, the second on psychometric testing, and the third on defining a cut-off score for “good performance.”

## Phase 1. Instrument design

### Step 1. Literature research and metrics screening

The first step of phase 1 involved developing a set of performance metrics. During a preliminary literature review, we identified 126 metrics from 27 studies, which two authors extracted into a Microsoft Excel ® spreadsheet (including details such as the name, brief description, standard, and measurement type). After identifying duplicates and removing unspecified metrics, 97 metrics remained. A consensus meeting was then held involving four research professionals from various specialties, such as clinical research coordinators, clinical research nurses (CRNs), research nurses, and nurse managers, all with over five years of experience in clinical research. A **CRN** focuses on patient-centered clinical trials, ensuring protocol adherence and participant safety, while a **Nurse Researcher** generates new knowledge and evidence-based standards to advance nursing practice. Involving both roles is crucial as CRNs provide practical insights from patient interactions, and Nurse Researchers contribute theoretical expertise, ensuring research is both clinically relevant and scientifically robust [11]. The main focus of the discussion was to identify which metrics posed the greatest challenges in daily practice to form the basis of a core set of metrics. In this phase, the decision to include or exclude metrics was discussed during the consensus meeting. The authors will need to agree on both inclusion and exclusion. Fleiss' Kappa, a statistical measure of inter-rater agreement for categorical variables, will ensure consistent metrics selection across the expert panel. A value of 0.70 or higher will indicate substantial agreement. As a result, 24 metrics will be included in the content validity phase.

### Step 2. Content validity

The preliminary version of the instrument will comprise the 24 items discovered in Step 1, categorized into three theoretical sub-scales (enrollment, data quality and management, protocol violations). The performance measures will undergo a critical evaluation to assess their relevance by a self-administered questionnaire distributed to 12–20 specialists in clinical research (e.g., clinical research coordinators, clinical research nurses, statisticians, nurse managers, clinical research associates). They will be requested to evaluate and quantify the validity (in terms of relevance) of the items both individually and collectively, propose improvements, and identify any omissions, as advised by Ayre & Scally [12]. Relevance will be validated using a 3-point scale, where 0 = “not essential”; 1 = “relevant”; 2 = “essential”.

Three open-ended questions will be posed to evaluate the consistency and clarity of the questionnaire. In instances of substantial discord among experts, issues will undergo review and modification through iterative conversations until a consensus is achieved.

## Phase 2. Psychometric testing

### Study design

Cross-sectional study.

### Setting

The study will be conducted across multiple research sites in Italy, including Institutes for Research, Hospitalization, and Healthcare (IRCCS), University Hospitals, and public hospitals involved in human experimentation.

### Inclusion and exclusion criteria

All ongoing and completed CTs will be included in the study to avoid selection bias.

### Variables collected

The researchers involved will collect key study identifiers, including variables such as study type, center, department (e.g., oncology, hematology, cardiology), treatment (e.g., single contact, multiple contacts), phase (e.g., Phase I, Phase II, Phase III, Phase IV) translational nature, trial design (e.g., RCT, Basket Trial, Platform Trial, Cluster Trial), presence or absence of special procedures, central processes, number of subjects enrolled, duration. Additionally, the involvement of a CRO (Contract Research Organization) and sponsor will be noted as either present or absent.

### Outcome assessor

The CT-SPM will not be completed by a single individual but rather by the entire study team. To ensure a thorough and accurate evaluation of site performance, all individuals who have worked directly on the trial or possess in-depth knowledge of its operations will be required to contribute to the completion of the instrument. This collaborative approach is essential to minimize individual bias and ensure that all performance aspects, including participant recruitment, data quality, and protocol adherence, are adequately assessed.

## Step 1. CT-SPM testing

Descriptive statistics will be performed for the demographic characteristics of the sample and items, including skewness and kurtosis indices, to assess normality. Construct validity will be evaluated using both exploratory factor analysis (EFA) and confirmatory factor analysis (CFA). A cross-validation approach will be employed, with the sample randomly divided into two sub-groups, selecting approximately 60 % and 40 % of the studies. An EFA will be conducted on the first sub-group (60 %) and a CFA on the second sub-group (40 %). The EFA will utilize the maximum likelihood method, with the selection of factors guided by eigenvalue analysis and the scree test. Items with a loading value exceeding 0.30 will be retained. Prior to conducting the EFA, Bartlett's test and the Kaiser–Meyer–Olkin (KMO) index will be examined to determine the factorability of the correlation matrix. Internal consistency will be assessed using McDonald's ω coefficient. McDonald's ω is preferred over Cronbach's alpha for assessing the reliability of a multifactorial construct because it does not assume tau-equivalence, meaning that all items contribute equally to the construct [13]. Omega provides a more accurate estimate of reliability when items have different factor loadings [13]. CFA will be used to cross-validate the most plausible factor structure model identified through the EFA. The evaluation of the CFA model will include the following fit indices: omnibus fit indices such as chi-square (χ²), incremental fit indices such as the Comparative Fit Index (CFI; values > 0.95 indicating a good fit), the Root Mean Square Error of Approximation (RMSEA; values < 0.06 indicating a good fit), and the Standardized Root Mean Square Residual (SRMR; values <1.0 indicating a good fit). Stability will be assessed by measuring reliability through the test-retest approach. To measure test-retest reliability, outcome assessors in the sample were asked to complete the instrument for 20 CTs [14] twice, 48 h apart. Intraclass Correlation Coefficients (ICC) estimates and their 95 % confidence intervals were calculated based on a mean-rating (*k* = 10), absolute-agreement, two-way mixed-effects model. Values above 0.75 indicate good test-retest reliability [15]. Spearman/Pearson's r, ANOVAs, and χ² tests were used to examine the interrelations between total scores and the other variables collected after accepting normality. All statistics will be calculated using R 4.3.3. [16] and Mplus Version 8.1 [17].

## Step 2. CT-SPM short form (SF)

To ensure ease of use across diverse CT sites, a short form will be developed, focusing solely on the most essential and universally applicable performance metrics. Mokken scaling, a nonparametric IRT model, was chosen for its ability to create hierarchical scales that maintain robust internal consistency while reducing the overall number of items. This approach allows researchers to develop questionnaires with multiple items to evaluate health-related components efficiently. In this study, a larger sample of professionals involved in clinical research will rate each metric on a 5-point Likert scale, reflecting the frequency with which each metric is encountered in practice (1 = ‘not frequent’, 5 = ‘highly frequent’).

### Statistical analysis

MSA consists of two elements: a) an automated procedure for item selection that categorizes a collection of ordinal items into Mokken scales and b) techniques for examining assumptions of nonparametric item-response theory (IRT) models [18,19]. Mokken models are based on three fundamental assumptions: unidimensionality, local independence, and latent monotonicity [18]. Unidimensionality refers to the property of a scale where it assesses only one underlying characteristic, referred to as the latent characteristic θ. Local independence refers to an individual's response to a specific question being unaffected by their responses to other items within the same assessment. Latent monotonicity states that the probability of a certain response level for each item rises in a non-decreasing fashion with the latent trait θ. The Monotone Homogeneity Model (MHM) postulates that the elements within a scale are unidimensional, exhibit monotonicity, and display local independence. If these conditions are satisfied, responders can be ranked based on the total score obtained from the items (3). The double monotonicity model (DMM) possesses the attribute of item-item ordering (IIO), in addition to the qualities of the MHM. IIO stands for items that maintain the same “difficulty ordering” regardless of the latent trait's value. The arrangement of items is determined by their level of difficulty, indicating whether they are challenging (rare) or less challenging (common). It is essential to develop a scalable hierarchy that can be consistently replicated across various samples. To begin with, we shall verify the assumption of unidimensionality by employing Loevinger's scalability coefficients H. The scalability coefficient (H) quantifies the extent to which an item may effectively rank individuals based on the degree of the construct being measured by the test. An item with a high scalability coefficient can accurately differentiate individuals with varying degrees of the attribute, whereas an item with a low scalability coefficient may not be able to sufficiently distinguish between these individuals. The coefficients will consist of three indexes: item-pair (Hij), item (Hi), and scale (Hs) scalability coefficients. If the value of Hs is equal to 1, then the scale will be classified as a perfect Guttman scale. The MHM will specify that these three indices must be within the range of 0 to 1. Higher H values will indicate a greater item discrimination capability. The next part will delineate the criteria for Loevinger's scalability coefficients: A scale is deemed weak if the value of H is between 0.3 and less than 0.4. It is termed moderate if H is between 0.4 and less than 0.5. A scale is considered robust if H is equal to or exceeds 0.5. Items with inadequate H values and/or those that violate the IIO will be eliminated from the scale during the IIO process, which seeks to reduce the item count. The Molenaar-Sijtsma ρ reliability metric will be utilized to assess internal consistency. Next, violations related to monotonicity (coefH) will be evaluated. Monotonicity pertains to the degree of consistency in ordering items on a scale along the underlying dimension that the scale aims to assess. For instance, if a scale exhibits monotonicity, it implies that scores will continuously rise as the difficulty or severity of the scale's components increases.

## Sample size calculation

In this study, sample size determination was conducted through a power-based simulation to ensure sufficient power to detect meaningful differences in MSA, which typically requires a larger sample size compared to EFA and CFA [18,19]. The parameters set for the initial simulation included a baseline of 24 items, an initial sample size of 200, and 100 simulations per iteration. The desired statistical power was set at 0.80, with a minimum H coefficient of 0.4, across a maximum of 20 iterations. During the simulations, the average power was calculated after each set of 100 simulations. If the estimated power was below the desired level of 0.80, the sample size was incrementally increased by 10, and the simulations were rerun. This iterative process continued until the estimated power reached at least 0.80 or the maximum number of iterations was achieved. The results from these simulations indicated that achieving the desired power with the given parameters and effect size would require a sample size between 400 participants by employing 20 iterations and 600 employing 40 iterations. These sizes were reached by adjusting the iterations and observing the outcomes at each step.

## Phase 3. Defining performance cut-off

In the study, the item “Overall performance” will be measured dichotomously, with “0” indicating that the protocol proceeded as expected, and “1” indicating that the protocol deviated from the expected course. To calculate a cut-off on this dichotomous measure, the metrics will first be standardized by converting their original Likert scale (1–5) into a 1–100 scale. The cut-off for “good performance” will then be determined using Receiver Operating Characteristic (ROC) curves, allowing for the identification of the optimal threshold to distinguish between adequate and non-compliant performance. ROC curves were chosen to determine the optimal cut-off because they provide a robust method for evaluating the trade-off between sensitivity and specificity, ensuring that the dichotomous performance measure is precise and reliable. Additionally, the Area Under the Curve (AUC) will be calculated to assess the overall discriminative power of the model in distinguishing between the two performance categories.

### Sub-groups analysis

If feasible, sub-group analyses will be performed to further refine the cut-off and ensure its applicability across different types of clinical trials. The sample will be divided into sub-groups based on variables such as trial phase (e.g., Phase I, II, III, IV), department (e.g., oncology, hematology, cardiology), trial type (e.g., RCT, Basket Trial, Platform Trial), and the presence or absence of special procedures or central processes. ROC curves and AUC values will be calculated separately for each sub-group to examine if the cut-off threshold varies between different types of trials or settings. This approach will help determine whether a universal cut-off is appropriate or if tailored thresholds are necessary for specific trial characteristics. Additionally, this analysis will assess how consistently the cut-off performs across various trial settings, providing insights into the reliability and robustness of the defined threshold for “good performance” in different clinical research environments.

### Protocol validation

*Not applicable.*

## Limitations

While the development of the CT-SPM marks a significant step toward standardizing site performance evaluation, several limitations must be considered. First, the assessment of clinical trial sites is inherently influenced by the interpretation and judgment of the evaluators using the initial iteration of the CT-SPM-SF. Although the tool is designed to standardize evaluations, variability in how individual evaluators perceive and score certain metrics—particularly those involving subjective criteria such as data quality or protocol adherence—may lead to inconsistencies. Training evaluators can help mitigate this variability. Second, the study is conducted across a range of clinical trial centers in Italy, which may restrict the applicability of the findings to different healthcare systems and CT environments, especially in countries with different regulatory frameworks or trial management structures. Future validation of the CT-SPM in international settings is recommended to ensure broader applicability.

## CRediT authorship contribution statement

**Mattia Bozzetti:** Conceptualization, Methodology, Writing – original draft. **Rosario Caruso:** Conceptualization, Methodology, Supervision, Writing – review & editing, Resources. **Silvia Soncini:** Conceptualization, Writing – original draft. **Monica Guberti:** Conceptualization, Methodology, Supervision, Writing – review & editing, Resources.

## Declaration of competing interest

The authors declare that they have no known competing financial interests or personal relationships that could have appeared to influence the work reported in this paper.

## Data Availability

No data was used for the research described in the article.
